# Decision-making and evidence use during the process of prenatal record review in Canada: a multiphase qualitative study

**DOI:** 10.1186/s12884-015-0503-6

**Published:** 2015-03-31

**Authors:** Sonia Semenic, Nancy Edwards, Shahirose Premji, Joanne Olson, Beverly Williams, Phyllis Montgomery

**Affiliations:** School of Nursing, McGill University, 3506 University Street, Montreal, Quebec H3A 2A7 Canada; University of Ottawa, 1 Stewart Street, Ottawa, Ontario K1N 6 N5 Canada; Faculty of Nursing, University of Calgary, 2500 University Drive NW, Calgary, Alberta T2N 1 N4 Canada; Faculty of Medicine, University of Calgary, TRW Building, 3rd Floor, 3280 Hospital Dr. NW, Calgary, AB Canada; Faculty of Nursing, University of Alberta, Edmonton Clinic Health Academy (ECHA), 11405 – 87 Ave, Edmonton, Alberta T6G 1C9 Canada; School of Nursing, Laurentian University, Ramsey Lake Road, Sudbury, Ontario P3E 2C6 Canada

**Keywords:** Prenatal care, Prenatal records, Guidelines, Evidence utilization, Decision-making, Context, Population health, Prenatal smoking, Prenatal alcohol use

## Abstract

**Background:**

Prenatal records are potentially powerful tools for the translation of best-practice evidence into routine prenatal care. Although all jurisdictions in Canada use standardized prenatal records to guide care and provide data for health surveillance, their content related to risk factors such as maternal smoking and alcohol use varies widely. Literature is lacking on how prenatal records are developed or updated to integrate research evidence. This multiphase project aimed to identify key contextual factors influencing decision-making and evidence use among Canadian prenatal record committees (PRCs), and formulate recommendations for the prenatal record review process in Canada.

**Methods:**

Phase 1 comprised key informant interviews with PRC leaders across 10 Canadian jurisdictions. Phase 2, was a qualitative comparative case study of PRC factors influencing evidence-use and decision-making in five selected jurisdictions. Interview data were analysed using qualitative content analysis. Phase 3 involved a dissemination workshop with key stakeholders to review and refine recommendations derived from Phases 1 and 2.

**Results:**

Prenatal record review processes differed considerably across Canadian jurisdictions. PRC decision-making was complex, revealing the competing functions of the prenatal record as a clinical guide, documentation tool and data source. Internal contextual factors influencing evidence use included PRC resources to conduct evidence reviews; group composition and dynamics; perceived function of the prenatal record; and expert opinions. External contextual factors included concerns about user buy-in; health system capacities; and pressures from public health stakeholders. Our recommendations highlight the need for: broader stakeholder involvement and explicit use of decision-support strategies to support the revision process; a national template of evidence-informed changes that can be used across jurisdictions; consideration of both clinical and surveillance functions of the prenatal record; and dissemination plans to communicate prenatal record modifications.

**Conclusions:**

Decision-making related to prenatal record content involves a negotiated effort to balance research evidence with the needs and preferences of prenatal care providers, health system capacities as well as population health priorities. The development of a national template for prenatal records would reduce unnecessary duplication of PRC work and enhance the consistency of prenatal care delivery and perinatal surveillance data across Canada.

## Background

Prenatal medical records have long been considered important tools for the clinical management of obstetrical care, in addition to being vehicles for quality assurance, compensation and medico-legal documentation [1]. As a clinical tool, prenatal records contain prompts and care guidelines for a wide range of physical and psychosocial health indicators over the course of pregnancy, and thus can play a critical role in facilitating the translation of research evidence into obstetrical practice [2,3]. Prenatal records typically include a comprehensive baseline prenatal health history form, risk assessment tools, and additional forms or flow sheets for on-going documentation of care during prenatal visits and childbirth [4]. The records are usually transferred from prenatal care settings to the patient’s labour and delivery unit in late pregnancy, and in some settings, the advent of electronic prenatal records has facilitated timely sharing of patient information across obstetrical care providers [5]. Similar to integrated care pathways [6], prenatal records function as both clinical guide and documentation system for prenatal risk factors and interventions, and have been described as “probably the best developed charting system available in medical practice” [4]. Whereas care pathways are typically developed to translate established guidelines for specific conditions into local clinical management protocols [7], prenatal records include a wide range of clinical parameters and are often designed for universal application to state or provincial populations. In addition, prenatal records serve as population health screening tools and sources of data for perinatal surveillance systems, and allow for quality assurance monitoring of compliance with patient care standards [1].

In Canada, health care is constitutionally under the authority of provinces and territories. Maternity care delivery models thus vary across Canada in accordance with local resources, regulatory, educational, and population characteristics [8]. However, each of Canada’s jurisdictions (i.e., 10 provinces and three territories) has adopted the use of standardized prenatal records as a mechanism for setting universal prenatal care standards, enhancing communication of patient health information across the perinatal continuum as well as monitoring prenatal risk factors at the population level. In the absence of a national body responsible for perinatal care, 10 jurisdictions have developed their own prenatal record tools and have a government-mandated expert committee responsible for reviewing and updating their prenatal record (the remaining three use prenatal records developed in a neighbouring province/territory). As a result, prenatal records differ in content and format across Canada. We previously found that prenatal records from different Canadian jurisdictions varied markedly in their inclusion of assessment questions and intervention guides related to prenatal smoking [9] and alcohol use [10], despite the availability of evidence-based national guidelines for addressing these modifiable risk factors for adverse maternal and infant health outcomes [11,12]. This raised the broader question of how prenatal record committees (PRCs) accessed and integrated research evidence, particularly around complex social issues such as maternal substance use. Maternal smoking and drinking rates have been decreasing in Canada [13] but remain significant public health concerns given their related risks of perinatal morbidity and mortality as well as longer-term impacts on maternal and child health [14,15].

Despite the widespread adoption of standardized prenatal records in maternity care, literature on prenatal records is virtually non-existent. The only paper found comparing general prenatal record content (dated 1991) revealed that most American prenatal records contained items of traditional medical-obstetrical significance (e.g., medical history, fundal height), as opposed to more “contemporary” issues such as patient education and social risk factors such as smoking and drinking [1]. No studies could be found describing how prenatal records are actually developed, or what decision-making processes may account for variations in their inclusion of research evidence for routine prenatal care. Literature examining the development of other types of decision aids or guidelines for routine prenatal care is also scarce. An Australian study found that local protocols for routine prenatal care varied widely across institutions, were not consistent with national policies or research evidence, and rarely included content on maternal smoking despite good evidence for the effectiveness of smoking cessation interventions [16]. Similarly, a comparison of national prenatal care guidelines from four countries (including Canada) found little consistency in care recommendations within or across countries [17]. The authors attributed variations in the guidelines to differences in the purposes of the guidelines, differences in local health care systems, and the primary use of expert opinion rather than research evidence for guideline development [17].

Broader literature on the development of clinical management tools acknowledges that guideline recommendations are influenced not only by scientific evidence but also by clinical experience, expert opinion, patient preferences and feasibility [18-20]. Group structure and processes have also been found to influence judgments during the development of tools such as clinical practice guidelines (CPGs) and integrated care pathways. These include group size, composition and interpersonal dynamics; availability and quality of the research evidence; group members’ skills in evidence appraisal; and personal values, experiences and interests [20-24]. Furthermore, the development of clinical tools that double as legal records of care needs to take into account how the tool will impact charting practices, communication of information and practitioner accountability [6]. Studies of evidence use during the development of local care pathways have highlighted the tension of applying standardized guidelines that will be held as benchmarks of care to complex conditions demanding clinical judgement and individualised patient care [6,25]. On the other hand, decisions about care guidelines intended for more universal or public health application involve broader health policy considerations such as health system costs and resources, political priorities and the needs and values of the population [26,27]. As Canadian prenatal records combine guideline and documentation functions for both clinical and population health applications, they offer a unique set of documents to examine how clinical and population health considerations converge or diverge in this arena of complex decision-making.

We found only one study that examined decision-making processes used during expert group development of clinical guidelines for population-wide application. This study, by Dobrow and colleagues, yielded a conceptual framework for “context-based evidence-based decision-making” to identify contextual influences on evidence use in the “shift from an individual-clinical to a population-policy level” [28,29]. Their framework distinguishes between a modifiable internal context within which the decision-making process occurs and a broader, more fixed external context within which expert group decisions are to be applied. The internal decision-making context encompasses factors related to the purpose of the decision-making activity, group participants and processes used to arrive at decisions. The external decision-making context includes epidemiologic features of the health issue being addressed, extra-jurisdictional factors (e.g., experiences in other jurisdictions that may help inform decision-making), and political factors such as socio-economic and health system issues that may influence decision options [29]. According to this process-oriented model, both internal and external contextual factors impact on how evidence is weighed to justify decisions across the three stages of evidence utilization (i.e., the introduction, interpretation and application of evidence [28]). Although Dobrow and colleagues used their framework to study how provincial-level expert groups formulated health policy recommendations for cancer screening [28,29], the framework was intended to enhance understanding of evidence-based decision-making in the broad arenas of health and social policy [28].

As prenatal records play a central role in guiding and monitoring the delivery of prenatal care in Canada, it is critical that the decision-making processes underlying their content are made more transparent. This paper describes a multiphase, knowledge translation project that aimed to 1) describe and compare how prenatal records are reviewed and updated across different Canadian jurisdictions; 2) identify contextual factors influencing decision-making and evidence use during the process of prenatal record review; and 3) formulate recommendations for optimizing the prenatal record review process in Canada.

## Methods

**Figure 1:**
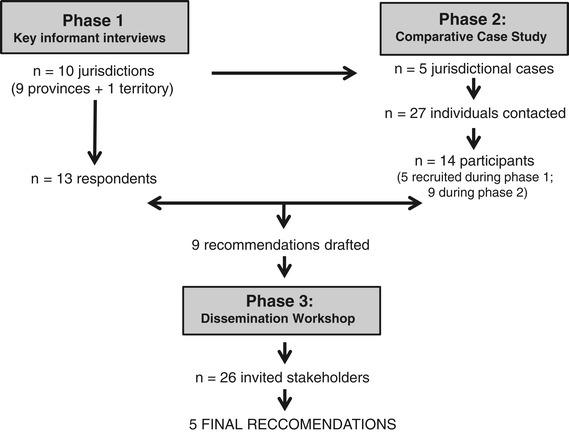
**Overview of project phases and participants.**

The project involved two data collection phases and a dissemination phase (see Figure 1). Phase 1 comprised descriptive key informant interviews with prenatal record committee (PRC) leaders across 10 Canadian jurisdictions. Phase 2 involved a qualitative comparative case study of PRC decision-making in five of the 10 jurisdictions. Phase 3 consisted of a dissemination workshop with key stakeholders to review project recommendations arising from phase 1 and 2 data analysis. Ethics approval for the project was obtained from the Research Ethics Boards at each of the study co-investigators’ affiliated university (i.e., University of Ottawa, McGill University, University of Calgary, University of Alberta and Laurentian University). All interviewees in Phases 1 and 2 provided written informed consent prior to their participation. Phase 1 and 2 interviews were conducted between June 2008 and March 2009, and the dissemination workshop was held in March 2010.

### Phase 1: Key informant interviews with PRC leaders

The main research question for Phase 1 was: *How are prenatal records developed, reviewed and updated across Canada*? We conducted in-depth, semi-structured interviews with a purposefully-selected sample of PRC leaders (i.e., chairs, co-leads or coordinators) from all 10 Canadian jurisdictions that had developed their own prenatal record. PRC leaders whose names were in the public domain were recruited directly via telephone and/or e-mail; the others were solicited via letters or e-mails forwarded through the organizational bodies responsible for prenatal record review. Interview questions were informed by Dobrow and colleagues’ conceptualization of the internal decision-making context and explored PRC views of the purpose of prenatal record review; PRC membership, mandates, and resources available to conduct the committee’s work; methods used to access and review research evidence; and strategies used for obtaining committee consensus on proposed modifications to the prenatal record.

Interviews were conducted in English via telephone by two trained research assistants except for two conducted in French by SS. Interviews were audio-taped and transcribed verbatim; French transcripts were professionally translated into English prior to analysis. Descriptive content analysis of the transcripts was conducted to summarize the prenatal record review process within each jurisdiction and to identify commonalities and differences across jurisdictions. Two investigators (JO, BW) reviewed the Phase 1 transcripts and extracted all pertinent information related to the main interview questions. The findings were then summarized and tabulated in a matrix, and double-checked for accuracy by a research assistant who reread all the Phase 1 transcripts. We also retrieved paper copies of each jurisdiction’s prenatal record as well as any accompanying user guides or assessment forms, to enable comparisons of specific content across jurisdictions and to facilitate understanding of interviewee’s descriptions or explanations related to the use of the form.

### Phase 2: Comparative case study of prenatal record decision-making

The main research question for Phase 2 was: *What internal and external contextual factors influence evidence use during the prenatal record revision process?* We used a qualitative multiple-case design with embedded units of analysis [30] to allow for in-depth examination of contextual factors influencing PRC decision-making. The “cases” were PRCs from diverse jurisdictions, and the “unit of analysis” was how and why PRCs made decisions about prenatal record content. We focused specifically on items related to maternal smoking and alcohol use as these address important predictors of infant health, vary across Canadian prenatal records [9,10] and represent controversial care issues that we hoped would reveal decision-making challenges. Based on the data obtained in Phase 1, we purposely selected five PRCs that varied in structure and review processes as well as the extent to which their jurisdiction’s prenatal record incorporated evidence-based recommendations for maternal smoking and alcohol use. We considered integration of research evidence on maternal smoking to be “weak” if their prenatal record simply contained a yes/no question to assess current smoking status, and “strong” if the record contained additional questions addressing past smoking history or exposure to second-hand smoke, provided for on-going monitoring of smoking and/or prompted referral to smoking cessation resources. Similarly, integration of research evidence related to maternal alcohol use was considered weak if the record only contained a yes/no question for current alcohol use, and strong if the record further assessed drinking patterns or past drinking history and/or included a standardized alcohol screening tool such as the T-ACE (Tolerance Annoyance Cut Down-Eye Opener) questionnaire [31]. Cases 1, 2 and 3 had prenatal records with strong items for both smoking and alcohol use; Case 4 had a record with strong smoking but weak alcohol use items, and Case 5’s record had weak smoking but strong alcohol use items. This matrix allowed for comparisons of PRC decision-making and evidence use both within and across the five cases.

The primary sources of data for the case study were in-depth, semi-structured interviews with members of the five selected PRCs, as well as any pertinent data about the PRCs’ decision-making contexts gleaned during the phase 1 interviews. All PRC leaders recruited in Phase 1 from the five selected cases were invited to participate in the Phase 2 interviews. Other eligible participants were any core or ad-hoc PRC members who participated in the jurisdiction’s most recent prenatal record review. These PRC members were recruited directly via telephone and/or e-mail (when their names were publically available) or via letters or e-mails forwarded through their PRC’s organizational body or committee chair. Information about our study was also e-mailed to potential participants through professional colleagues, and via the Society of Obstetricians and Gynecologists of Canada (SOGC). All Phase 2 interviews were conducted in English via telephone interviews by trained research assistants; audiotaped; and transcribed verbatim. Guided by the framework for context-based evidence-based decision-making, the Phase 2 interview explored how, and what types of evidence (research or other) were considered as part of committee decision-making around prenatal record content during the jurisdiction’s most recent review; factors influencing PRC access to research evidence as well as their decisions to apply (or not) this evidence; and broader contextual or health system factors that influenced PRC decision-making around prenatal record content. We inquired about how PRCs made decisions around maternal smoking and alcohol use items, as well as prenatal record content more generally.

Phase 2 interview data were analysed using both deductive and inductive content analysis techniques [32]. We developed a preliminary coding framework based on Dobrow and colleagues’ conceptualization of the internal and external decision-making contexts. Interview data were reviewed and coded under the corresponding categories, and open-coding was used to capture any emerging categories related to evidence use during PRC decision-making. A graduate student coded all transcripts and created a memo summarizing salient reflections of each transcript. SS double-coded 40% of the transcripts to evaluate coding accuracy and refine the coding framework. Next, findings across participants from the same PRC were synthesized and integrated with any relevant data from Phase 1 into a detailed summary of decision-making processes for each case PRC. Finally, data were extracted into summary matrices to compare internal and external contextual factors influencing PRC decision-making across the five cases [33].

### Phase 3: Dissemination workshop

Based on our findings from Phases 1 and 2, we drafted a set of nine recommendations with supporting rationales for enhancing the integration of evidence during the prenatal record review process across Canadian jurisdictions. We then held a full-day invitational symposium with key stakeholders to review and vet these preliminary recommendations. Invited participants included perinatal health researchers, policy-makers and association leaders from across Canada; as well as experts in public/population health, health surveillance and evidence-informed decision-making. We presented an overview of the project and its main findings, and provided each participant with a document containing the draft recommendations and their rationale. Participants were asked to rate each recommendation and supporting rationale for clarity, coherence and importance, on a seven-point Likert scale, and to provide suggestions for any modifications. Participants also engaged in small group work, facilitated by members of the research team, to discuss selected recommendations in more depth, consider strategies for implementation and provide suggestions for reframing recommendations with an action-orientation. Key points from the small group work were then shared among all participants, and used to shape our project’s final recommendations.

## Results

### Phase 1: Prenatal record committee structures and processes

We were able to identify and recruit the chair or coordinator of the PRC in each of the 10 Canadian jurisdictions that had developed their own government-mandated prenatal record. In three of these jurisdictions, two different PRC members in leadership roles were interviewed, for a total of 13 key informants.

The 10 PRCs varied considerably in their composition, mandates and review processes. Groups responsible for prenatal record review across Canada included provincial-level perinatal health or reproductive care programs (PH/RCPs), perinatal committees within provincial medical associations or perinatal branches of the jurisdiction’s Ministry of Health. The PH/RCPs typically conducted prenatal record reviews as a funded deliverable for their Ministry of Health. PRCs housed within medical associations were chaired exclusively by a physician whereas all the others were co-led by a physician and nurse or coordinated by a nurse consultant/manager. The PRCs varied in size from five to over 15 members and were composed primarily of physicians representing a variety of perinatal providers (e.g., obstetricians, general practitioners, perinatologists), selected for their clinical expertise and/or positions within local professional organizations or government agencies. Other clinician members included nurses, nurse practitioners, midwives and nutritionists. Only a few PRCs included non-clinicians such as consumer representatives or data management specialists. However most PRCs sought ad hoc feedback from a broad range of stakeholders to help review or pilot updated versions of the prenatal record forms, including experts consulted for particular health topics, front-line workers using the prenatal records, representatives from special interest/advisory groups, and health record specialists.

Four of the 10 PRCs were standing committees that met on a regular basis (e.g., as part of a provincial-level perinatal committee) whereas the others typically convened their PRC only in response to accumulating user demands for revisions to the form. Although none of the key informants identified any formal mandates concerning how often their prenatal record should be reviewed, most reported that they conducted informal reviews of their prenatal record at least annually and organized a more extensive review of the prenatal record every three to five years. Whereas most of the 10 jurisdictions had updated their prenatal records within the past three years of the study interviews (or were in the process of doing so), two had not made any modifications to their prenatal record in more than 10 years. Primary reasons given for initiating their most recent prenatal record review were to include more content related to the determinants of health (e.g., stress, smoking, alcohol use, domestic violence) and to integrate new national guidelines related to such topics as genetic screening and maternal serum screening.

Only three PRCs used formal procedures for systematically collecting and reviewing the research evidence during their most recent prenatal record review process. Research evidence was typically supplied to the PRCs by individual members or solicited from external experts as needed. Few PRCs had access to support persons such as librarians or research assistants to help retrieve the scientific literature. None of the PRCs used formal procedures to obtain consensus on modifications to the prenatal records; most key informants reported that committee agreements were achieved through informal debate and group discussion. Only two jurisdictions provided honoraria or financial reimbursement for committee participation. Overall, PRCs managed by PH/RCPs appeared to have the most infrastructure support (via access to program administrative staff and funds for meetings) as well as more extensive links with colleagues and other stakeholders for external consultation. Decision-making appeared least inclusive when reviews were conducted by small perinatal committees within medical associations.

### Phase 2: Contextual influences on PRC decision-making and evidence use

Recruitment of participants for Phase 2 interviews proved difficult due to a lack of public information on PRC membership in the five jurisdictions selected for the case study, as well as the often transient, ad hoc nature of the PRCs. We were able to contact 27 PRC members across the five cases, out of approximately 40 potential participants (the five selected PRCs varied in size from five to 12 members). Of those reached, 14 (52%) agreed to participate (including five committee leaders who also completed the Phase 1 interviews). As most of the PRC members approached were busy clinicians, refusal to participate in the study was mainly due to “lack of time”. A total of 14 participants completed the Phase 2 interview (3 committee members each for four of the cases and two members for the remaining case). Of the 14 participants, the majority (n = 11) were women, and represented the professions of medicine (n = 7), nursing (n = 6) and midwifery (n = 1). The majority (n = 11) worked primarily as clinicians and had been practising in their profession for a range of 6 to 40 years. Participants had been involved in their jurisdiction’s PRC for a range of 2 to 16 years.

The selected PRCs were from geographically diverse jurisdictions, including two western provinces, two eastern provinces and a northern territory. The PRCs varied according to their type (ad hoc vs. standing committee); affiliated organization, size and membership composition (Table 1).

**Table 1:** **Prenatal record committee type, size and composition**

	**Case 1**	**Case 2**	**Case 3**	**Case 4**	**Case 5**
**Committee type and organizational affiliation**	Ad hoc prenatal record working group convened and chaired by PH/RCP coordinator	Standing joint planning committee of the PH/RCP, co-chaired by physician and ministerial population health representative	Ad hoc committee of health ministry-appointed Maternal Perinatal Committee, coordinated by perinatal nurse-consultant and chaired by an obstetrician	Ad hoc prenatal record working group convened by PH/RCP, co-chaired by obstetrician and perinatal nurse consultant	Standing perinatal and maternal mortality committee of the provincial medical association, chaired by an obstetrician
**Committee size**	Large (10–15 members)	Large (10–15 members)	Small (5–10 members)	Small (5–10 members)	Small (5–10 members)
**Membership composition**	PH/RCP coordinator, GP representative from the provincial medical association, medical experts (e.g., GP, obstetrician, perinatalogist), nurse, midwife, electronic health record expert, data management specialist, experts in aboriginal health	PH/RCP coordinator, population health specialist, medical experts (GPs, obstetrician, pediatrician, reproductive care specialist), acute care nurse, community health nurse, dietician, consumer representatives data management specialist	Perinatal nurse-consultant, obstetrician, GP, midwife, community health nurse, representative from aboriginal women’s health program, medical officer of health	Obstetrician, perinatal nurse consultant, neonatalogist, GP, perinatal clinic nurse, acute care nurse	Obstetricians, GPs, pediatricians, neonatologists, representative from the provincial nurse’s association

PH/RCP: provincial perinatal health/reproductive care program; GP: general practitioner; ObGyn: obstetrician/gynecologist.

Participants from all five PRCs confirmed that maternal smoking and alcohol use items were reviewed during their most recent prenatal record review, and that research evidence (e.g., literature reviews, primary studies) was used to help inform their decisions about whether or not to modify the items. However, the PRCs differed in how they obtained, interpreted and applied the research evidence (Table 2) as well as the extent to which other sources of evidence (e.g., expert opinion) influenced their decision-making. Most PRCs added questions to improve assessment of both maternal smoking (e.g., past smoking history, exposure to second-hand smoke) and alcohol use (e.g., T-ACE questionnaire). In contrast, the two PRCs with “weak” items decided to retain a single yes/no question to assess current smoking (Case 5) or alcohol use (Case 4) following their evidence review. Cross-case analyses pointed to the interaction of a number of internal and external contextual factors influencing PRC decision-making and evidence use.

**Table 2:** **Prenatal record committee processes for the introduction, interpretation and application of research evidence**

**Stages of evidence use**	**Case 1**	**Case 2**	**Case 3**	**Case 4**	**Case 5**
**Introduction** (Who supplied the research evidence?)	PH/RCP coordinator, PRC members; expert consultants, researcher with RA support, clinician colleagues	PH/RCP coordinator, expert consultants, individual PRC members	PRC chair, PRC member with summer student support, expert consultants from Ministry of health	PH/RCP coordinator, expert consultants, clinician colleagues	3-member PRC sub-committee, with librarian support
**Interpretation** (How was the quality of the research evidence appraised?)	Use of evidence hierarchies to evaluate research evidence	PRC members trusted the expertise of those supplying the research evidence; literature considered “high quality” if published in peer-reviewed journals and adopted by other jurisdictions	Primary reliance on synthesized sources of evidence; PRC accepted research evidence as valid if integrated by other jurisdictions into their prenatal records	PRC members trusted/assumed that each had expertise in evaluating research quality	PRC members trusted the expertise of those supplying the research evidence
**Interpretation** (Who else was consulted for advice or feedback on proposed revisions to the prenatal records?)	Other provincial perinatal agencies, substance abuse specialists, university researchers, public health specialists, clinicians selected to review proposed revisions	Experts on specific topics (e.g., substance abuse), prenatal record committee members in other jurisdictions, clinician stakeholders via provincial professional groups, professional organizations represented on the committee, academics	Clinician stakeholders (nurses, physicians, midwives), local native women’s councils, nurse management group, prenatal record members in other jurisdictions, Ministry of Health experts in health promotion and substance abuse, electronic record specialist	Provincial perinatal advisory committee, colleagues from provincial tobacco and alcohol strategies, clinician stakeholders (nurses, nurse-practitioners, MDs, midwives) from across the province	Suggestions for revisions solicited from obstetricians and GPs through provincial professional associations and university departments of medicine (obstetrics, pediatrics, family practice)
**Application** (How was final consensus on revisions to the prenatal record obtained?)	Committee informally aimed for consensus; agreement to maintain a clinical focus as the priority; “bargaining” with final decisions based on consensus among members from the different physician groups, sometimes the “loudest voice wins”.	Agreement that prenatal record needed to reflect “best practices”; negotiation of what worked best for the majority; commitment to persist with the review until everyone “can live with the product”.	Agreement through “good discussion” until consensus was reached; seeking a compromise between needs of physicians, midwives and nurses (e.g., length of the form); physician dominance of the decision-making process.	Consensus reached through discussion and then consulted widely outside the committee; established priorities to manage the volume of information collected for the review process; made “executive decisions” in the face of contradictory feedback from external consultations.	Longstanding committee with high levels of mutual respect among members; consensus reached through discussion but “not everyone had to agree”; members may concede their opinion if a respected colleague felt strongly about a proposed revision.

PH/RCP: provincial perinatal health/reproductive care program; RA: research assistant.

### Internal decision-making context

Internal contextual factors (i.e., decision-making purpose, participants, processes) that influenced evidence use by PRCs included resources available for evidence reviews; group composition and dynamics; perceived function of the prenatal record; and reliance on expert opinion.

#### PRC resources

The PRCs were commonly constrained by a lack of time, resources and/or skills from conducting a critical review of the scientific evidence for all the content areas of the prenatal record. The PRCs typically relied on synthesized sources of research evidence such as systematic reviews and best practice guidelines (particularly those from the SOGC), which were interpreted in light of their jurisdictional context:

*“We are influenced by professional associations who have the resources really to do an extensive review of a particular topic… it doesn’t mean that we just take that information at face value - we also look at the reference information and research information that they’ve used to make their decision and we also look at the local context and see how that can be incorporated”* (Case 4).

The two large PRCs affiliated with provincial perinatal care programs had the most infrastructure support for evidence reviews and appeared to have a more explicit mandate for evidence-informed decision-making: *“There was a recognition that we needed to use the evidence that was presented, that we needed to use best practice*” (Case 2). Conversely, members from the smaller PRCs acknowledged that the size of their committee limited reviews of the available literature: *“alcohol fell through the cracks…the evidence really wasn’t brought forward for full discussion”* (Case 4). However, smaller PRCs were also regarded as more efficient due to the volume of decisions that had to be made about prenatal record content: *“The working group isn’t as effective if it gets larger…it takes more time to get things through”* (Case 3).

Only one of the five PRCs used formal strategies (i.e., evidence hierarchies) to evaluate the quality of research evidence (Table 2). The others typically trusted that their members were supplying robust evidence or had the skills to evaluate evidentiary quality: *“It (research evidence) would be brought forth and accepted pretty much de-facto…”* (Case 5). Faced with limited resources, one small PRC also *“piggy-backed”* on the work done in other jurisdictions, assuming other PRCs had more capacity to conduct evidence reviews:

*“We sort of looked at the prenatal forms from various jurisdictions and basically cherry picked what we thought would be important and relevant for our communities…I don’t think we looked very hard at, you know, how good the evidence or what the quality of the evidence was. We figured that if it had been included in other jurisdictions then it must be good”* (Case 3).

Given the burden of evidence reviews, PRC members from several cases suggested having a national initiative to facilitate updating of their prenatal records rather than having to “reinvent the wheel” during each review:

*“The problem for example, of alcohol consumption, isn’t unique to [Province 1] or even all of Canada…I think that there would be room for supporting the application of evidence for each of the provinces by some national group…so that the research does not have to be done on every question locally in 10 provincial jurisdictions”* (Case 5).

#### PRC composition and dynamics

A preponderance of clinical experts (primarily physicians) and relative absence of methodologists on the PRCs contributed to a focus on the usefulness, acceptability and feasibility of research evidence, rather than debates over evidentiary quality: *“…committee members were experts within their own field…you know, specialists, general practitioners so what they did is responded to the recommendations based on what they thought was practical”* (Case 1). All five PRCs used informal means (i.e., group discussion) to arrive at consensus about revisions to the prenatal records (Table 2), which may have truncated full consideration of decision options: *“Umm, it’s basically the one who speaks the loudest…and if there is agreement by everyone else…there is no formal process for that kind of thing. It was whether the change made sense…”* (Case 1). In some PRCs, this allowed decision-making to be influenced by professional hierarchies among group members: *“…the majority of changes were made by a small elite group of people, you know, the obstetricians and a few specialists and physicians with very little input from other people…”* (Case 5).

PRC composition also influenced the nature of the evidence brought to the decision-making table, in addition to power dynamics. Participants from two PRCs noted that their committee was under-represented by nurses and midwives, given their expanding role in the delivery of prenatal care. Only one PRC involved patient representatives, who were seen as adding a *“very helpful layer”* to practical deliberations of prenatal record content: *“…sometimes they have a perspective of, ‘Well this seems very reasonable, I know, it’s because I’ve had a baby*” (Case 2). Two PRCs invited experts in aboriginal care on their PRC to *“ensure questions related to issues such as substance abuse were asked in a sensitive manner…political-wise it was very important to be seen that we were approaching the (native women’s groups) and getting information from them”* (Case 3).

#### Purpose of the prenatal record

PRC views of the primary function of prenatal records determined the focus of the review process and what types of evidence were considered in decisions about prenatal record items. Members from all five PRCs reported that the main purpose of the prenatal record was to guide and communicate clinical care:

*“So one of the things that we’re looking at in our prenatal program, is how we’re going to be able to collect some of the variables on the prenatal record for further analysis and research and program planning. But in that light, we’re very, very cognizant that first and foremost we need it to be a clinical tool and a communication tool and it’s not there for data mining… Ensuring the committee has a clinical focus as its number one priority just has to be a principle”* (Case 1)*.*

The importance of the prenatal record as a communication tool was recognized in particular by PRCs in jurisdictions with multiple (i.e., shared care) or more transitory, rural prenatal care providers:

*“We wanted [the prenatal record] to collect as much, what we thought would be useful information and do it in such a way that when we had such a transitory healthcare provision…that it would be relatively easy and straightforward to get this information and to pass it on”* (Case 3).

The prenatal record’s priority function as a clinical (rather than population health) tool was also reflected in the relative absence of population health and data management experts on the PRCs. Nonetheless, in several cases PRC decision-making about the specific content of the prenatal record forms took into account the prenatal record’s current or future role as a source of data for perinatal health surveillance:

*“Over the last few years we’ve been trying to get up a perinatal database, look at outcomes and all various different factors. And I think that played a big part in some of the questions that we sort of wanted to ask… I mean we even looked at second hand smoking as, is this relevant data to be collecting?”* (Case 3).

Participants described the challenge of ensuring that prenatal record questions were appropriate for both individual patient care and population health monitoring, noting the need for *“a more planned and integrated approach…with the same wording in the prenatal record and the maternal child data base”* (Case 4). Clinical and population-health functions of the prenatal record sometimes required different types of information, highlighting the complexity of decision-making around the specific items:

*“Our [Public Health] individuals wanted to use what primarily was reported in the literature for indicating morbidity such as number of drinks per week. And yet our clinical individuals would say, for us on the intervention perspective, the number of drinks per week is not the issue; the issue is, is there any alcohol consumption and what’s the maximal alcohol consumption on any one occasion”* (Case 1).

Poor compliance with prenatal care provider documentation of such “sensitive” questions as smoking or alcohol use was noted as a particular problem for perinatal data base managers:

*“I mean smoking is always a challenging variable to look at…so we’re constantly trying to improve the data based on what we get when we search for things, and also based on what the coders actually tell us they’re able to collect”* (Case 4).

PRCs that were developing electronic versions of their jurisdiction’s prenatal record faced the additional challenge of matching paper and electronic copies of the forms. Nevertheless, participants acknowledged that electronic prenatal records facilitated information-sharing among clinicians, expanded opportunities for linking with perinatal databases and created possibilities for tailoring forms to the particular monitoring needs of individual patients.

#### Reliance on expert opinion

PRCs resorted to expert opinion when research evidence needed to inform decisions about modifying items (e.g., aetiology of the risk factors, intervention effectiveness) was viewed as negative, equivocal or lacking; or was in conflict with personal opinions or experiences of committee members. For example, one small PRC decided not to add more assessment questions or intervention guides for maternal smoking based largely on members’ beliefs that such strategies were ineffective, despite the availability of strong research evidence to the contrary:

*“There was some significant discussion on the committee about this [incorporating suggestions about smoking cessation], and the reason is the rather dismal performance of most of the attempts to try and secure smoking cessation. I mean unfortunately, it’s all very well to identify smokers, but there’s not a whole lot that has been demonstrated to work that results in smokers decreasing their smoking even during pregnancy*” (Case 5).

Another participant from the same PRC noted that relying on a few volunteers to research the literature was a potential source of bias: *“When we try and get evidence to make these kinds of decisions, we are doing it each as individuals and there’s no question that there is a subjective angle to that…we pick and choose things that we like…”* (Case 5). Decision-making around items related to alcohol use appeared particularly complex for the PRCs due to mixed research findings about the impact of social drinking:

*“There was some discussion around the alcohol question, ‘cause there certainly is, you know, different opinions in the literature around the effects of alcohol, and how much alcohol can cause damage. But there certainly was agreement that certainly you would want to be promoting ‘no alcohol’ as best”* (Case 1).

### External decision-making context

External contextual factors (i.e., epidemiological, extra-jurisdictional, political) that influenced PRC decisions about prenatal record content included concerns about user buy-in; health system capacities; and pressures from public health stakeholders. Table 3 summarizes participant descriptions of public health features related to prenatal smoking and alcohol use as well as prenatal health services in their jurisdiction.

**Table 3:** **Jurisdictional context related to prenatal smoking/alcohol use, and prenatal health services**

	**Case 1**	**Case 2**	**Case 3**	**Case 4**	**Case 5**
**Prevalence of prenatal smoking and alcohol use**	Rates of maternal smoking/alcohol use are high among the jurisdiction’s aboriginal population	Rates of maternal smoking/alcohol use are lower than in other jurisdictions, but PRC feels this may be due to under-reporting	Large aboriginal population with high rates of maternal smoking/alcohol use	Rates of maternal smoking are high	Rates of maternal smoking/alcohol use are high among the jurisdiction’s aboriginal population; increase in aboriginal birth rate has led to increased population rates of FASD
				PRC assumes rates of maternal alcohol use are lower than in other jurisdictions	
**Key public health features related to maternal smoking and alcohol use**	Jurisdiction has a strong tobacco control lobby	Recent local public health awareness campaigns related to maternal smoking, alcohol use and FASD	Social drinking in pregnancy is common and culturally accepted among some groups	Jurisdiction has a strong tobacco control lobby	Active local public health awareness campaigns related to maternal smoking, alcohol use and FASD
	Strong cultural values against drinking alcohol during pregnancy				
			Tobacco use and FASD prevention are priority public health issues		
**Prenatal health service**	Prenatal care delivered by GPs and obstetricians, with growing trend towards shared/collaborative maternity care with allied health professionals (midwives, nurses)	Prenatal care delivered primarily by obstetricians (midwifery not legislated)	Prenatal care delivered primarily by nurses, midwives	Prenatal care providers vary in skill levels, particularly in rural areas	Prenatal care delivered primarily by physicians, but nurses increasingly becoming the first point of entry of pregnant patients
	Jurisdiction is developing electronic prenatal health records	Jurisdiction has a perinatal database	Large turn-over of health care provider workforce	Midwifery in the process of being legislated	Midwifery in the process of being legislated
	Jurisdiction has a perinatal database		Prenatal record is integrated into electronic health records	Jurisdiction has a perinatal database	
			Jurisdiction in process of developing a perinatal database		

FASD: fetal alcohol spectrum disorder.

#### User buy-in

PRCs considered the acceptability of prenatal record items to the clinicians who would be completing the forms, given resource constraints within the practice setting: *“Right now (the form) sits with 72 questions that you’re supposed to ask in the first (prenatal) interview. Well, that’s not humanly possible…”*(Case 1). Several participants noted that a more comprehensive, detailed prenatal record based on the latest evidence might not receive buy-in from prenatal care providers:

*“…a committee themselves can be sold on the evidence and the requirement for information on the prenatal record. The challenge would be how you best sell that to the practitioners when you’re implementing the record because of the reaction to the increased time and resources required to interview the mother and document the information and then knowing what to do with that information”* (Case 1).

To address potential resistance to change, all five PRCs solicited feedback from clinician stakeholders on proposed revisions to the forms, through individual consultations or small-scale piloting of the revised prenatal record forms. Participants from four PRCs also stated that they explicitly aimed to keep their prenatal record short and “user-friendly”, necessitating committee debate about the relative importance of prenatal records items: *“The page was already pretty full, so if we wanted to add something, we thought well we don’t want a multi-page form because people won’t use it… what can we eliminate so that we can add some stuff?”*(Case 5). Considerations of item relevance involved weighing evidence for efficacy against considerations of feasibility given the PRC’s jurisdictional context:

*“If I think back to the smoking one…it was space on the paper if I remember correctly, and you know, “And is there a value in doing it?” … “Is it a ‘nice to know’, or a ‘need to know’?” And is it applicable in this practice setting? Is it a big issue?”* (Case 2).

Several participants raised the particular issues of clinician discomfort addressing complex social issues such as prenatal substance abuse, and high rates of non-compliance with documentation of maternal smoking and alcohol use items:

*“When we talked to people who were using the current prenatal record, and wondering why these particular points were not sort of not being checked off or asked about…they either felt uncomfortable about the question or they didn’t know what to do if the [answer to the] question was positive…”* (Case 5).

One PRC created a self-report questionnaire as a companion document for their prenatal record, to facilitate collection of personal information: *“We found that sometimes the physicians were uncomfortable about asking those questions…so we introduced the [X] questionnaire, which is completed by the mother herself* (Case 1). Members from two PRCs with large aboriginal populations and high rates of maternal substance use also addressed the acceptability of the prenatal record items to pregnant women, acknowledging the potential negative impact of substance use questions on patient-provider trust, patient privacy, and child protection involvement:

*“So if there was concern by the mother that this information [substance abuse question]…may lead to her social service referral, or child welfare referral and her loss of the child…it certainly could impact on how she answers the questions.” (Case 3).*

#### Health system capacities

PRC decisions about the applicability of evidence also considered the availability of health system resources to implement prenatal record recommendations at the population level: *“Social economically…we had to be very aware on the committee of what resources were available in the big center, as well as the smaller centers” (Case 3).* Clinician skills and the availability of local smoking cessation or addiction services were important health system considerations when debating whether to include more screening questions or intervention prompts related to smoking/alcohol use. For example, Case 4 added items to prompt a more detailed assessment of smoking patterns given their confidence that health system resources were available for referrals: *“…supports are provided throughout the province where people can get support and information and smoking cessation aids for free so that we could tag into that information when we’re supporting people to reduce or quit smoking…”* Yet they decided not to add a screening tool for alcohol use partly in consideration of the lack of alcohol treatment programs for pregnant women in their jurisdiction: *“Probably the biggest challenge with all of that for care providers is…figuring out what do we do now when someone says, “Yes, I’m using alcohol,” and the approach to that and the resources…”*

#### Pressures from public health stakeholders

Local public health priorities and external pressures from public health stakeholders or community groups were additional influences on whether to modify prenatal record items and what questions to prioritize in the competition for prenatal record space: *“There was quite a vocal group from the African Canadians saying we weren’t paying enough attention to things like Sickle Cell”* (Case 4). PRCs from jurisdictions with high aboriginal populations were under particular pressure to improve prenatal record screening for maternal substance use:

*“I mean we do have very high rates of smoking, very high rates of alcohol abuse and substance abuse here in [X], for a small population. And there’s always been outside pressure to…ask the right questions and provide interventions that would help that. A lot of government and non-government sort of organizations do give their input into what we should ask, or why we should be asking these questions”* (Case 3).

In several cases, the public health trend towards harm reduction approaches to substance use influenced the specific content and wording of smoking and alcohol use items:

*“And the smoking issue, I can’t remember what research we had but…like the old prenatal record just said, you know, how many cigarettes you have in a day. This time it was more like we have to take a look all the time at harm reduction so how much were they smoking before they got pregnant”* (Case 4).

For the case that opted not to enhance their prenatal record’s alcohol screening questions despite evidence-based recommendations, the PRC ultimately considered whether the limited prenatal record space would better serve more pressing public health priorities:

*“There were things like the T-ACE screen brought forward for looking at people’s addiction, that type of thing. I remember taking a look at some of those research things, but decided that in [Province X] these are the things right now with this prenatal record, we need to focus on obesity”.*

### Phase 3: Development of study recommendations

Nine members of the research team and 26 invited stakeholders participated in the dissemination workshop. Invitees included five representatives of national professional associations (including medicine, nursing and midwifery); six stakeholders from different branches of Canada’s federal health agency or national maternal child health organizations; three representatives from national or provincial perinatal health surveillance programs; and several nationally-known perinatal or health promotion researchers. Based on group discussions, participants’ written feedback and ratings of the draft recommendations, we modified and synthesized the original nine recommendations for strengthening the prenatal record review process into five main messages. In brief, our recommendations address: involving multi-disciplinary stakeholders in the revision process; adopting the explicit use of decision-support tools, and processes to support the revision process; developing a national template of evidence-informed changes that can be used by provinces; considering both clinical and surveillance functions of the prenatal record; and developing a dissemination plan to communicate changes to the prenatal record. The recommendations and related key points derived from the interview and dissemination workshop are summarized in Table 4.

**Table 4:** **Recommendations for promoting the integration of research evidence into Canadian prenatal records**

**Recommendation**	**Related key points from project interviews and dissemination workshop**
*1) Ensure the involvement of multiple stakeholders in the development and review of prenatal records and in final decision-making about revisions to these forms. All disciplines that provide prenatal care should be represented to provide essential input on feasibility.*	Prenatal record forms serve the needs of diverse disciplines in addition to prenatal care providers with differing levels of skill (e.g., residents, rural practitioners with few pregnant patients). PRCs benefited from participation and feedback of a broad range of stakeholders, either through direct involvement on the review committees, consultation/feedback from stakeholders throughout the revision process, or piloting of the record prior to its finalization. Key stakeholders may include representation from a variety of clinical disciplines; members with expertise in electronic records/data management/health surveillance; research/methodology experts to support the formal evaluation and application of research evidence; population health experts to ensure that population health needs are taken into account when revisions are considered; patients/consumers for ensuring acceptability and relevance of prenatal record content; and health economists/policy-makers to address health system resources needed for the delivery of evidence-based prenatal care.
*2) Adopt formalized and explicit use of decision-support tools, decision-making processes and consensus approaches for the introduction, interpretation and application of research evidence in the development and revision of prenatal records.*	A thorough, non-biased review of the research evidence requires access to the extant literature requires, technical expertise and sufficient time. PRCs need to be appropriately resourced to support the participation of all relevant stakeholders in the review process. More formalized consensus procedures would make the prenatal record review process more transparent and help to enhance the process of record revision by:
	1) ensuring that the opinions of less “powerful” or vocal committee members are given equal consideration;
	2) making explicit what should be done in cases where research evidence and pragmatic clinical or health surveillance considerations are at odds;
	3) providing a formalized mechanism for decision-making about issues for which the research evidence is equivocal.
*3) Consider both clinical and surveillance functions of the prenatal record form and appropriately use evidence to support both functions.*	Different types and sources of evidence and evidence synthesis approaches are required for these two complementary data collection functions of the forms, including: risk factor etiology, effectiveness of assessment strategies to identify risk factors, effectiveness of interventions to address risk factors and alternative approaches to enhance/support clinical providers, and population health implications of not addressing a risk factor. Prenatal record committee composition needs to reflect these complementary but distinctive core functions of the form. Prenatal record data standards need to be aligned with national surveillance and existing perinatal health indicators.
*4) Develop an evidence-based, national template for a prenatal record, to facilitate/promote adoption of optimal standards of evidence-based prenatal care across Canada.*	A national prenatal form template that reflects best research evidence would help decrease unnecessary duplication of work across prenatal record committees in different Canadian jurisdictions; support consistent prenatal care when patients move across jurisdictions; and elicit more uniform data for a national perinatal surveillance system. A national template should be available in a flexible format that can be adapted/tailored to the particular needs and context within each jurisdiction. Development of a national template should involve representatives from all Canadian jurisdictions and key stakeholder organizations.
*5) Plan and adequately resource comprehensive, effective and tailored strategies for dissemination of prenatal record modifications (e.g., outreach education; development of supporting guides to describe and provide a rationale for changes to the forms).*	Insufficient support to busy clinicians using the revised prenatal record forms may contribute to provider frustration, dissatisfaction with the revised forms, and lack of compliance with completion of the forms. Disseminating revised prenatal record forms along with a guiding document that clearly points out all changes and their rationale was an important strategy used by many jurisdictions.

## Discussion

This is the first study we are aware of to examine decision-making processes underlying the content of standardized prenatal records. Our use of Dobrow and colleague’s framework for context-based evidence-based decision-making [28] helped elicit a broad range of internal and external contextual factors influencing evidence utilization by PRCs and revealed the complexity of decision-making around prenatal record content. Our findings illustrate how PRCs sought to reconcile the competing functions of the prenatal record as a comprehensive clinical guide for a range of health indicators, a legal documentation tool, and a data source for perinatal health surveillance. Research evidence related to effective screening and intervening for maternal smoking and alcohol use was weighed against non-research evidence such as professional experiences and opinions, practical concerns about the length of the prenatal record forms, perceptions of prenatal care provider workload and skills, the availability of local referral resources, and public health pressures. Consistent with Dobrow and colleague’s description of how expert groups developed population-based cancer screening recommendations [28], non-research evidence ultimately guided PRC decisions about the appropriateness and feasibility of implementing evidence-based screening recommendations within their jurisdictions. However the specific ways in which PRCs prioritized these diverse or conflicting considerations, and how consensus was reached on final decisions about what to include on the prenatal record forms, remains unclear.

In our findings and proposed recommendations, we highlight several important issues related to the development and updating of prenatal records. First, input from all relevant stakeholders and the use of systematic strategies for reviewing the extant literature and guiding group decision-making would help reduce potential biases in the prenatal record review process. We found little documentation of PRC membership or processes available in the public domain, raising questions about the transparency and/or accountabilities of PRCs across Canada. The PRCs we examined were clinician-dominated with sometimes little (or no) representation or supports from methodology experts to help evaluate research evidence, or other persons who could advise on other functions (e.g., surveillance) of the prenatal record. Previous studies of evidence use by clinical guideline groups have found that clinician-led decision-making favours professional values, expert opinion, and practical experience over research evidence [20,22,24,34,35]. Our study is also consistent with previous findings suggesting that group composition and professional hierarchies influence how guideline recommendations are formed [20-23], reinforcing the importance of attending to small group processes that may limit the contribution of minority or less-powerful group members. Representative stakeholder involvement along with processes to support constructive debates of the evidence would ensure more valid interpretation of the research evidence within the wider clinical and social context, as well as help PRCs negotiate the competing functions of the prenatal records in a way that maximizes benefits to patient and population health outcomes. Although our findings suggest larger, more inclusive committees were better equipped to review the available evidence, participants also mentioned the need to balance group size with efficiency. Eccles and colleagues noted that the desire for wide representation in guideline groups needs to be weighed against the need for a cohesive working group, and suggested between eight to ten members as optimal for a small group [36].

Findings underscore the importance of adequate resourcing of the work of PRCs, to allow for a systematic evaluation of the updated literature and adequate time for consultation and decision-making processes. Larger-scale guideline initiatives that involve stakeholders from practitioners to policy makers require sufficient time, commitment and resources to ensure effective communication with all concerned parties and to support their implementation [34]. We found surprisingly little infrastructure support for the prenatal record review process in some Canadian jurisdictions, particularly for the technical help needed to identify and synthesize research evidence. Studies of guideline development processes have found that time and resource constraints may limit discussions of research quality in favour of discussions of more pragmatic issues [37,38]. Our finding that resource-limited PRCs adopted prenatal items from other jurisdictions under the assumption that they were well-researched attests to the potential usefulness of a national process to define standards and items for prenatal records, based on the best available evidence that could then be applied across Canada. There is a need to identify optimal strategies for monitoring and updating prenatal records (e.g., full versus partial updating) [39,40] given the burden and duplication of PRC efforts across Canada. However, the potential for a national prenatal record raises other questions such as how PRCs would take contextual considerations into account when they review primary and synthesized sources of research evidence versus when they are using evidentiary-based guidelines proposed by others.

An additional key issue raised by our study was the relative lack of attention paid to the potential population health implications of prenatal record items, such as how they could be used to lever improvements in wider health care system characteristics (e.g., better referral systems for women with addictions) that might enhance the health of future pregnant women and infants. Although providing better care for population sub-groups such as Aboriginal women was identified as a consideration by some PRCs, none of the participants expressed PRC intentions to lobby for improvements to the health care system. Rather, limitations of the care system were identified as reasons for not making changes on the prenatal record forms. Perhaps this orientation of PRCs is related to a perception of their mandate, the large amount of work involved in reviewing many items, and the mix of items on the forms (some with more obvious implications for patient-provider interactions, others with wider implications for the health system).

PRC composition and decision-making around prenatal smoking/alcohol use items revealed a primary focus on the prenatal record as a tool for individual clinicians. The use of prenatal records as a legal documentation tool with the potential implications of missing data for clinician accountability may have accounted in part for PRC reluctance to include recommendations that were not feasible within the context of local health care resources [25].

In contrast to concerns about prenatal record use by clinicians, the role of the prenatal record as an important data source for perinatal health surveillance and the consequences of absent or poorly completed items for population health researchers (e.g., underestimation of the prevalence of smoking or high-risk alcohol use during pregnancy) appeared to be of less direct concern to PRCs. There also seemed to be a tension in deliberations about using the record for clinical versus population level goals. The issue of under-reporting of stigmatizing behaviors such as alcohol use during pregnancy is well-documented [41], and women’s disclosure of substance use may be more accurate if the data remains confidential and is used for research purposes without being attached to a clinical record. As electronic record-keeping improves, other options for meeting clinical and surveillance requirements of the prenatal record may be possible. For instance, more in-depth data about tobacco and alcohol use (or other risk factors) could be electronically captured and diverted for population health and surveillance use only.

Although not explicitly identified as ethical considerations by participants, several domains of ethical issues were raised during interviews: a) professional ethics, i.e., how the prenatal record forms might aid clinicians to meet their professional obligations; b) patient ethics, i.e., how the inclusion of items on the forms might assist clinicians to optimize benefits and minimize harms to patients; and, c) population health ethics, i.e., how characteristics of the population, varying patterns of service access across population sub-groups and underlying factors that influence these access patterns should be taken into consideration in decisions about modifications to prenatal record items. For example, the underlying ethical dilemma of minimizing harms may have been a consideration in the integration of more comprehensive assessment questions about maternal smoking and alcohol use. A key barrier for discussing and documenting alcohol use in the perinatal period is not only clinicians’ preoccupation with the dilemma of what to do with a positive answer (prompting the need to identify multilevel support and treatment for substance use and to engage in evidence-based and compassionate brief interventions), but also potentially adding to women’s burdens by asking them about something that is highly stigmatized and for which child protection may become involved [42]. The need for attention to such ethical matters in the review process raises the importance of explicitly integrating a discussion of these ethical dimensions into decision-making by PRCs.

There was a surprising absence of discussion about governance and financing models in the Canadian health care system, and how these affected PRC choices about items for inclusion on the prenatal record forms. A number of participants made reference to issues of time constraints, busy clinicians and provider mix, which their PRCs had considered in reviewing practical limitations to the addition of assessment questions. However, underlying health system drivers such as fee for service models were not addressed. Clinician reluctance to “open Pandora’s box” by asking about complex social issues during the limited time allocated to prenatal visits may have been an unspoken concern of the PRCs. Fee for service funding models may have broader implications for PRC discussion about pragmatics, the length of the prenatal record forms, trading off concerns about user buy-in with public health priorities and sources of clinician resistance to change. The potential influence of these more fundamental health system issues on the prenatal record review process needs to be made more transparent.

Finally, the impact of prenatal records as knowledge translation tools ultimately rests on individual clinician adherence to the record’s guidelines, which will vary according to such factors as their perceived relevance or degree of sanctions for non-compliance with documentation on the forms [43]. The inclusion of evidence-based items to the prenatal record does not ensure their application and we do not know how quickly new prenatal care requirements generated by revisions to the tools get integrated into clinical practice. As modifications to the prenatal records provide opportunities for both retrospective and prospective natural experiments, future studies are needed to examine the actual use of prenatal records in practice, and the impact of prenatal record items on perinatal health outcomes at both the local and population levels.

There were several methodological limitations to this study. The number of participants was small and while purposively sampled, may or may not reflect the perceptions of other committee members. We also examined PRC decision-making using a retrospective, cross-sectional case study design. In some jurisdictions the prenatal record review process had taken place months or years prior to our interviews, therefore some participants may have had difficulty recalling specific details of their committee’s decision-making process. There has also been a time lapse between the interviews and publication of our findings, and contextual factors related to PRC decision-making may have changed in some jurisdictions since our data collection (e.g., the adoption of electronic prenatal records). However, a re-review of all Canadian prenatal records conducted in January 2014 revealed that only one of the 10 Canadian jurisdictions involved in our project had modified their prenatal record content since 2009, and Canada still lacks a national process for prenatal record review. Future research should consider prospective designs using non-participatory observation of PRC decision-making [38,44], as well as a broader examination of how decisions are made about the varied content of prenatal record forms. For example, the translation of research evidence related to prenatal assessment of standard biomedical parameters such as maternal serum screening may be more straightforward. However, our findings related to prenatal screening for smoking and alcohol use exposed a range of factors that may be applicable to other complex and/or stigmatizing maternal risk factors such as maternal depression, conjugal violence, and healthy weight management. Many PRCs across Canada indicated that their most recent prenatal record review was driven in part by the need to include more content on the social determinants of health, suggesting the need for more guidance in this area.

## Conclusions

Universal prenatal records are potentially powerful tools for the translation of best-practice evidence into routine prenatal care, and thus merit rigorous processes for ensuring the quality of their content. Decision-making related to prenatal record content needs to consider the multiple functions of the tool, and involves a negotiated effort to balance evidence-based recommendations with the needs and preferences of prenatal care providers, health system capacities as well as population health priorities. Broad stakeholder participation, systematic evaluation of the research evidence and formal strategies for arriving at group consensus are needed to reduce potential biases in PRC decision-making. The development of a national template for prenatal records could reduce duplication of work by PRCs in different Canadian jurisdiction and enhance the quality and consistency of prenatal care delivery and perinatal surveillance data across Canada.

## Abbreviations

PRC: Prenatal record committee
PH/RCPs: Perinatal health or reproductive care programs
GP: General practitioner
ObGyn: Obstetrician/gynaecologist
FASD: Fetal alcohol spectrum disorder
RA: Research assistant
